# Identification of Grape Laccase Genes and Their Potential Role in Secondary Metabolite Synthesis

**DOI:** 10.3390/ijms251910574

**Published:** 2024-09-30

**Authors:** Hao Wang, Haixia Zhong, Fuchun Zhang, Chuan Zhang, Songlin Zhang, Xiaoming Zhou, Xinyu Wu, Vivek Yadav

**Affiliations:** The State Key Laboratory of Genetic Improvement and Germplasm Innovation of Crop Resistance in Arid Desert Regions, Key Laboratory of Genome Research and Genetic Improvement of Xinjiang Characteristic Fruits and Vegetables, Institute of Horticultural Crops, Xinjiang Academy of Agricultural Sciences, Urumqi 830091, China; wanghao4836@nwafu.edu.cn (H.W.); zhonghaixia1@sina.cn (H.Z.); zhangfc@xaas.ac.cn (F.Z.); 2016204005@njau.edu.cn (C.Z.);

**Keywords:** laccase, grapevine, gene family, secondary metabolites, RNA-seq, berry development

## Abstract

Laccase, a copper-containing oxidoreductase, has close links with secondary metabolite biosynthesis in plants. Its activity can affect the synthesis and accumulation of secondary metabolites, thereby influencing plant growth, development, and stress resistance. This study aims to identify the grape laccases (*VviLAC*) gene family members in grape (*Vitis vinifera* L.) and explore the transcriptional regulatory network in berry development. Here, 115 *VviLACs* were identified and divided into seven (Type I–VII) classes. These were distributed on 17 chromosomes and out of 47 *VviLACs* on chromosome 18, 34 (72.34%) were involved in tandem duplication events. *VviLAC1*, *VviLAC2*, *VviLAC3*, and *VviLAC62* were highly expressed before fruit color development, while *VviLAC4*, *VviLAC12*, *VviLAC16*, *VviLAC18*, *VviLAC20*, *VviLAC53*, *VviLAC60* and *VviLAC105* were highly expressed after fruit color transformation. Notably, *VviLAC105* showed a significant positive correlation with important metabolites including resveratrol, resveratrol dimer, and peonidin-3-glucoside. Analysis of the transcriptional regulatory network predicted that the 12 different transcription factors target *VviLAC*s genes. Specifically, WRKY and ERF were identified as potential transcriptional regulatory factors for *VviLAC105*, while Dof and MYB were identified as potential transcriptional regulatory factors for *VviLAC51*. This study identifies and provides basic information on the grape LAC gene family members and, in combination with transcriptome and metabolome data, predicts the upstream transcriptional regulatory network of *VviLAC*s.

## 1. Introduction

Laccase (*p*-diphenol: dioxygen oxidoreductase, EC 1.10.3.2) is a polyphenol oxidase containing four copper ions, which can catalyze a variety of substrates and redox to form water, and are widely present in bacteria, fungi, higher plants [1]. The physiological function of plant laccases is mainly involved in cell wall lignin synthesis, phenolic compound polymerization, and plant defense regulation [2,3,4].

Since the discovery of laccase in the sap of sumac trees in Japan, research on the function of laccase has attracted more and more attention. So far, the laccase gene family has been identified and characterized in *Arabidopsis thaliana* [5,6], cotton [7], rice [8], citrus [9], *Setaria viridis* [10], *Brassica napus* [11], and *Pyrus bretschneideri* [12]. In plants, laccase is involved in different biological processes, the earliest studies have shown that plant laccase is expressed in lignified tissues and can catalyze the polymerization of lignin precursors [1,4]. Arabidopsis *AtLAC11, AtLAC4*, and *AtLAC17* encode laccase genes that affect lignin levels [2,13]. The stem of *AtLAC4* and *AtLAC17* mutants showed moderately reduced lignin levels, while the stem of *lac4* and *17* mutants showed 40% less lignin [13]. This shows that the change in laccase activity in plants can also affect the degree of lignification of xylem tissue. Laccase can boost the oxidation process of a broad range of phenolic compounds [14,15]. For example, *AtLAC15*, also known as TRANSPARENTTESTA10 (TT10), has been shown to play a key role in the oxidative polymerization of flavonoids in the seed coat of Arabidopsis [16]. Later studies revealed that the extractable lignin content of *AtLAC15* mutant seeds decreased by almost 30% when compared to wild-type seeds. Additionally, the soluble anthocyanin or condensed tannins increased by 59% [17]. In addition, the findings of Liang et al. (2006), showed changes in seed coat permeability, seed germination, and root elongation in the mutant of Arabidopsis. This suggests that laccase may play a role in the growth and development process [17]. Recent studies have shown that plant laccase plays a crucial role in the degradation of anthocyanins. The unidentified ADE (anthocyanin-degrading enzyme) has now been identified as laccase. Its high expression in litchi pericarp is found to result in significant degradation of anthocyanins, a finding that has been further confirmed through transient expression in tobacco leaves [18].

Grapes are favored by consumers worldwide due to their nutritional value. According to statistics from the International Organization of Vine and Wine (OIV), the annual global output has reached 79 million tons (https://www.oiv.int/, accessed on 16 March 2023). Of this total, approximately 30% is used as table grapes, intended for direct consumption by consumers. Previous studies have indicated that laccase plays a significant role in secondary metabolite production. Additionally, grape berries are known to contain a high abundance of secondary metabolites. This has drawn our interest in exploring the potential connection between laccase and grape berries. The phenylalanine metabolic pathway includes the most typical lignin monomer synthesis pathway. Starting from phenylalanine, through a series of methylation, hydroxylation, reduction, and connection reactions, the above three types of lignin monomer with different methylation levels are formed, and finally, these monomers form phydroxyphenyl (H), guaiacyl (G), and syringyl (S) through oxidative polymerization, respectively, which are linked by many bonds to form lignin in the plant polymer [19]. Furthermore, many enzymes in the synthesis of lignin monomer play a catalytic role, the most important ones are L-phenylalanine ammonia-lyase (PAL), 4-coumarate coenzyme ligase (4CL), cinnamyl alcohol dehydrogenase (CAD), peroxidase (POD), and laccase (LAC) [20]. The effect of enzymes on lignin synthesis is self-evident. Studies have shown that inhibition of *GhLAC1* expression in cotton leads to metabolic redirection and accumulation of jasmonic acid and secondary metabolites, suggesting that laccase may be one of the factors affecting secondary metabolic synthesis [15]. In grapevine, it was reported that VvmiR397a and target VvLACs genes, including VvLAC4,7,11,14, and VvLAC17, play important role in berry development [21]. Lignin, which is regulated by laccase genes is also found to be responsible for russet disorder in grape berries [22] Lacasse genes have a close relationship with defense mechanisms in plants. For instance, the grape *LAC3* gene was identified to be a virulence determinant in *B. cinerea* disease [23]. However, the effect of laccase on other secondary metabolites (flavonoids, anthocyanins, etc.) in grapes is rarely studied.

In this study, we conducted genome-wide identification of grape laccase genes. We used bioinformatics methods to analyze 115 grape laccase genes, including phylogenetic analysis, gene structure and conserved domain analysis, chromosome location, and collinearity analysis. Additionally, the expression patterns of these genes were determined by combining RNA-Seq and RT-qPCR data. Since laccase is involved in the regulation of plant secondary metabolites [3,4,8,16,24,25,26], the objective of this study is to investigate its possible role in grapevine metabolite regulation. To achieve this, we analyzed metabolome data from grape development and correlated them with laccase gene expression data. Additionally, we predicted transcription factors that may target laccase, thereby further exploring potential regulatory relationships. This provides an important theoretical basis for understanding the synthesis of secondary metabolites of laccase in grape.

## 2. Results

### 2.1. The Genome of Grape Harbors an Expanded Laccase Gene Family with 115 Members

**Figure 1:**
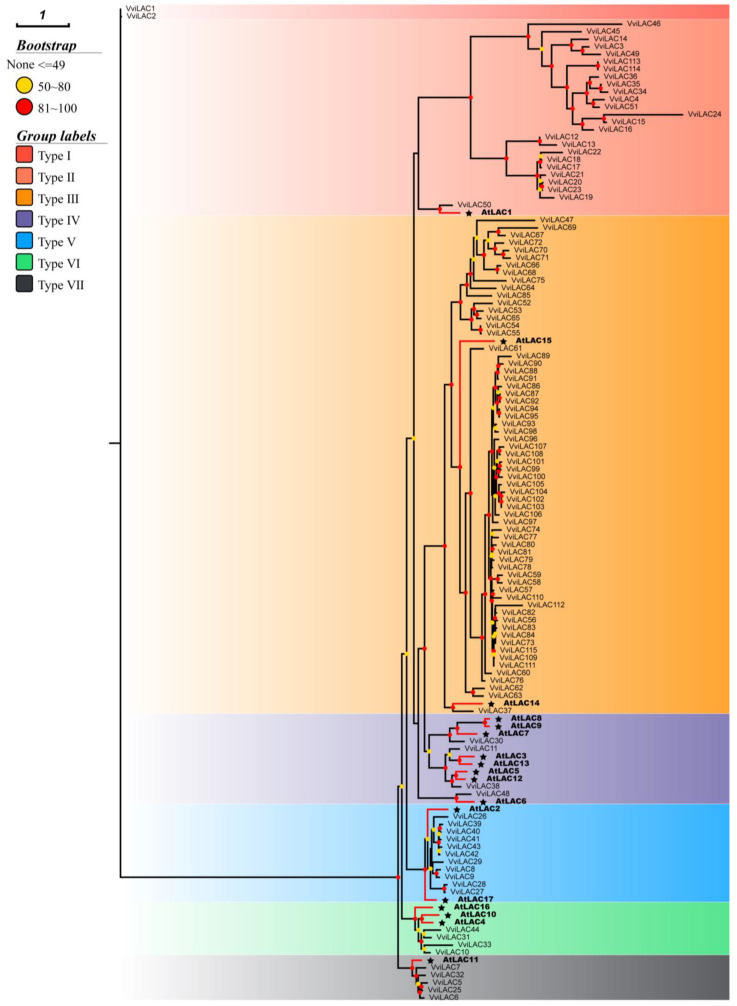
Phylogenetic analysis of laccase proteins in grape and Arabidopsis thaliana. The seven feature branches of the series are highlighted in different colors. Numbers at nodes indicate branch support performed by the ultrafast bootstrap test. Members of the Arabidopsis protein family are shown in bold and star symbols.

A total of 115 *VviLACs* were obtained, which were named *VviLAC1* to *VviLAC115* according to their position on the 17 chromosomes. Arabidopsis has 17 members of the laccase family, and phylogenetic analysis showed 6 branches. To further assess the phylogenetic relationships among Arabidopsis sequences, an amino acid-based phylogeny was constructed with the grape and Arabidopsis laccases based on the maximum-likelihood method (Figure 1). The resulting tree shows seven subclades, Arabidopsis laccase was observed in six branches, and the special group (type Ⅰ) contained only two *VviLAC* genes. Obviously, the distribution of grape laccase sequence among different groups was not uniform. Most strikingly, type III contains approximately 55.65% (64 out of 115) of *VviLAC* genes. Type II, type IV, type V, type VI, and type VII contain 25, 4, 14, 4, and 5 *VviLAC* genes, respectively.

The physicochemical properties of these 115 VviLACs were identified (Table S1). Their protein sequence length varied from 382 to 1153, the molecular weight ranged from 42.38~131.4 kDa, and the isoelectric point spanned 5.38 to 10.05. The online prediction for subcellular localization revealed that these genes are present in various cellular compartments, including the cytoplasm, extracellular space, chloroplast, and nucleus. However, a larger proportion of the genes were found in the cytoplasm and nucleus, while a smaller portion was localized in the extracellular and chloroplast regions.

### 2.2. Expansion of the VviLACs in Grape Was Mainly Caused by Tandem Duplications within Type III Genes

**Figure 2:**
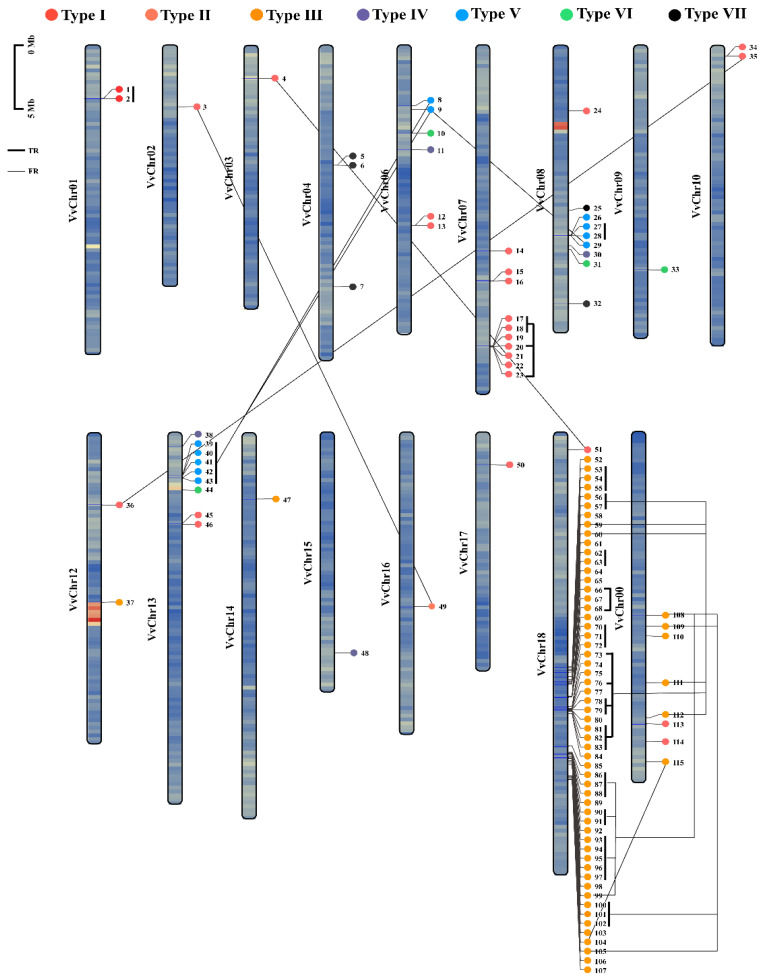
Distribution of laccase genes on the grape chromosomes. The circles with different colors at the top represent different classifications of VviLACs, the rectangle represents the chromosome, the left shows the chromosome number, and the internal color represents gene density, red: dense, blue: sparse. The scale of chromosome length is measured in MB. “▬”segments represent tandem repeats (TR), while “—” are connected to fragmented replication (FR).

To understand the molecular mechanisms underlying the expansion of the laccase family in grapevine, the genome distribution of all 115 *VviLACs* was determined by physically mapping them onto the 17 chromosomes (Figure 2). The analysis showed that the *VviLACs* are distributed on almost all chromosomes except chromosomes 5 and 6, but the distribution is uneven. A laccase gene is found on chromosomes 2, 3, 9, 14, 15, 16, and 17. Chromosomes 1, 10, and 12 all contain 2 *VviLACs,* and chromosomes 4, 6, 7, 8, 13, and 0 contain 3, 6, 10, 9, 9 and 8 *VviLACs*, respectively. The 57 *VviLAC* genes are located on chromosome 18 and are densely distributed. Through tandem replication analysis, it was found that about 72.34% (34 out of 47) of the *VviLAC* genes in chromosome 18 were involved in tandem replication events. The segment duplication results showed that about 65.22% (30 out of 46) of the *VviLAC* genes on chromosome 18 were involved in segment duplication events, and they all belonged to type III on chromosome 18, so type III caused grape laccase the driving force of gene family expansion.

### 2.3. Gene Structure and Conserved Motifs Analysis with 115 VviLACs

**Figure 3:**
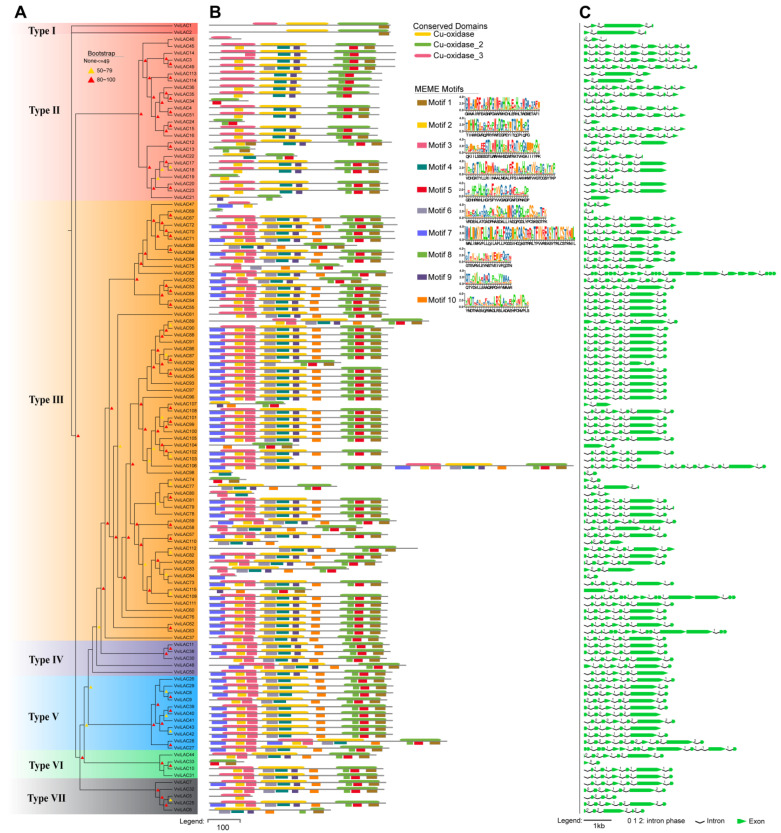
Phylogenetic relationships, the architecture of the conserved motifs and domains, exon–intron structures in grape laccase family members: (**A**) The phylogenetic tree was constructed based on the full-length sequences of grape laccase proteins using MEGA (version 7). (**B**) The conserved motifs of grape laccase proteins. Different colored rectangles indicate different motifs, which are numbered 1–10, and different colored bars indicate different domains, which are numbered 1–3. (**C**) Exon–intron structure of grape laccase genes. Green zones indicate coding sequence length (exon); black lines denote introns; and the number denotes the phases of the corresponding introns.

To better understand the similarity and diversity of VviLAC proteins in grape, we analyzed the phylogenetic tree (Figure 3A), conserved motifs (Figure 3B), and exon–intron structure (Figure 3C). The phylogenetic tree groups VviLAC proteins were conserved according to protein homology, gene structure, and motif distribution. VviLAC106 showed the longest genome sequence, containing 12 exons, while VviLAC69 showed the shortest genome sequence, containing only one exon. Three copper-binding domains are typical characteristics of most plant laccases. We also observed the same trend i.e., three domains in each identified grape laccase protein (Cu_oxidase, Cu_oxidase_2, and Cu_oxidase_3). A total of 10 motifs were identified, the domain information by PFAM corresponded and 10 motifs identified by MEME analysis to the upper and lower ends of the sequence line. Figure 3B shows that motifs 2, 3, and 7 were found in the conserved domain Cu-oxidase, similarly, motifs 4, 6, and 9 are in Cu-oxidase_2, and motifs 1, 5, and 8 are in Cu-oxidase_3.

### 2.4. Multi-Species Collinearity Analysis

**Figure 4:**
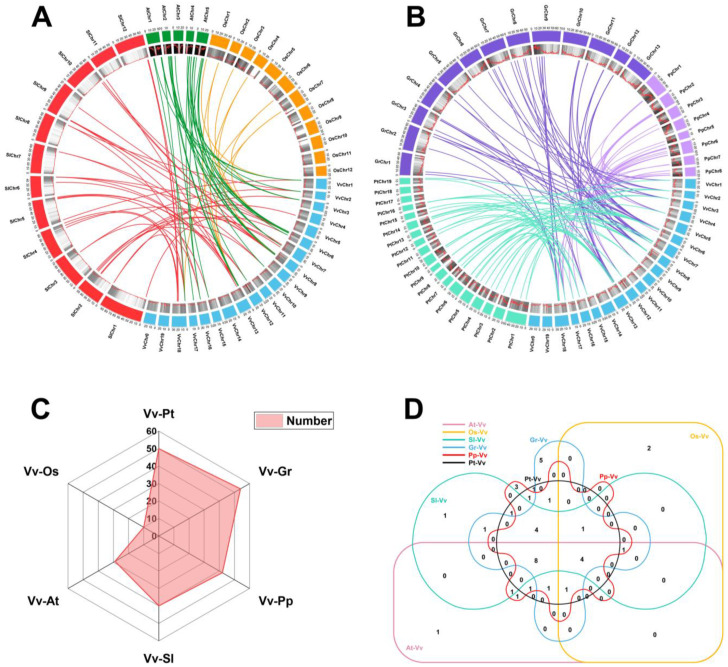
Synteny analysis of laccase genes between grape and six representative plant species: (**A**) Circle diagram of collinear relationships of grape (Vv), *Arabidopsis thaliana* (At), *Solanum lycopersicum,* (Sl) and *Oryza sativa* L. (Os) laccase genes. Different colored rectangles represent different species. (**B**) Circle diagram of collinear relationships of grape (Vv), *Populus trichocarpa* (Pt), *Gossypium raimondii* (Gr), and *Prunus persica* (Pp) laccase genes. (**C**) The number of homologous gene pairs between grape laccase gene and other species. (**D**) Relationship between the number of laccase genes homologous to grape and different species.

To further examine the phylogenetic mechanism of the grape laccase family, we identified the orthologous genes of the laccase gene family in grapes and other six representative plants, these include *P. trichocarpa*, *G. raimondii*, *P. persica*, *A. thaliana*, *S. lycopersicum,* and *O. sativa* (Figure 4A,B). Compared with monocot *O. sativa* (10 pairs), dicot plants and *V. vinifera* have more laccase orthologs, 29 pairs of Arabidopsis, 40 pairs of tomato, 42 pairs of *Prunus persica,* and *Populus trichocarpa* and *Gossypium raimondii* are 50 and 54 pairs, respectively (Figure 4C). Five direct homologous genes were identified in common between grapevine and six other species (Figure 4D). This indicated that these orthologous genes existed before their ancestors differentiated and played a key role in plants. In addition, eight grape laccase genes were found to have an orthologous relationship with five dicot species (Figure 4D). But they do not appear in monocot rice. This indicated that these gene pairs are produced after the differentiation of dicots and monocots.

Diverse functions were found by the GO analysis of VviLAC proteins (Figure S1). The majority of VviLAC proteins were involved in extracellular activities, whereas some of them were in the membrane part. Almost all VviLAC proteins had the molecular function of binding and catalytic activity. In terms of biological processes, VviLAC proteins participated in various biological pathways and regulated the various metabolic and biological processes, such as cellular process, metabolic process, response to stimulus, and biological regulation.

### 2.5. Expression of VviLACs during Grape Berry Development

**Figure 5:**
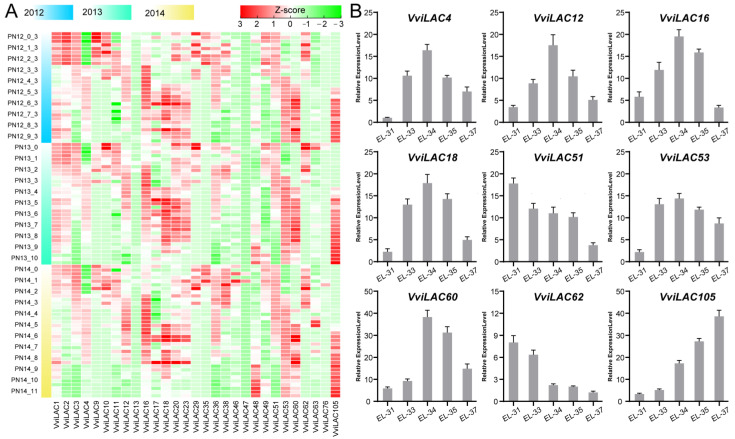
Expression heat map of *VviLACs* during grape berry development and qRT-PCR verification: (**A**) Expression of laccase gene during the development of grape berry sampled for three consecutive years. (**B**) qRT-PCR verification of laccase-related genes during the berry development stages of ‘Summer black’.

The differential expression of multiple laccase genes at different stages was found in the RNA-seq data of ‘Pinot Noir’ grape berry during the development and ripening stages. The expression profiling of *VviLACs* during fruit development was observed, and the FPKM of *VviLACs* was used to draw a heat map (genes with FPKM average < 1 were deleted). Figure 5A showed the expression of *VviLACs* genes during fruit development for three consecutive years (node No. 5 is the color transformation stage) in ‘Pinot Noir’. *VviLAC1*, *VviLAC2, VviLAC3*, and *VviLAC62* were highly expressed in early fruit development or before fruit color transformation. The expression of *VviLAC4, VviLAC12, VviLAC16, VviLAC18, VviLAC20, VviLAC53, VviLAC60*, and *VviLAC105* was higher in the middle and late stages of fruit development or after fruit color transformation. During the process of grape berry development, numerous secondary metabolites were synthesized and accumulated. This process also resulted in changes in the expression of certain *VviLACs*. The RT-qPCR test in ‘Summer black’ further demonstrated the changing trend of grape laccase genes during fruit ripening, which was consistent with the results of RNA-seq. In general, their expression may have a certain effect on the synthesis and accumulation of secondary metabolites in grape.

### 2.6. Relationship between Laccase and Metabolites in Grape Berry Development Stage

**Figure 6:**
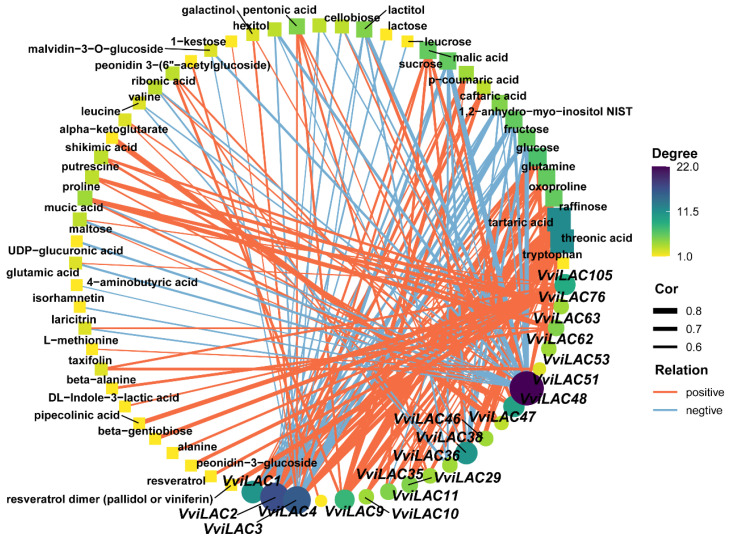
Correlation analysis between laccase gene expression and secondary metabolites. Line orange: positive correlation; Blue: Negative correlation. Circular nodes represent VvLACs, squares represent secondary metabolites, and node sizes are determined by network connectivity.

To investigate the effect of *VviLACs* on secondary metabolites of grape berries, correlation analysis was performed using digital RNA-seq and metabolome data of ‘Pinot Noir’ fruit at development stages [27]. As shown in Figure 6, *VviLAC1*, *VviLAC2*, *VviLAC3*, and *VviLAC51* were negatively correlated with carbohydrates, alcohols, phenols, acids, and flavonoids, while *VviLAC105*, *VviLAC62*, and *VviLAC9* were positively correlated with most metabolites. It is worth mentioning that *VviLAC105* had a significantly positive correlation with resveratrol, resveratrol dimer, taxifolin, and glucoside paeoniflorin chloride, while *VviLAC51* was significantly negatively correlated with malvidin-3-O-glucoside and isorhamnetin. In summary, it was found that *VviLACs* were significantly correlated with secondary metabolites such as flavonoids, phenols, sugars, and acids, suggesting that laccase may be involved in the synthesis and accumulation of multiple secondary metabolites (Table S2).

### 2.7. Predictive Analysis of Candidate Transcription Factors Targeting Laccase Family Genes

**Figure 7:**
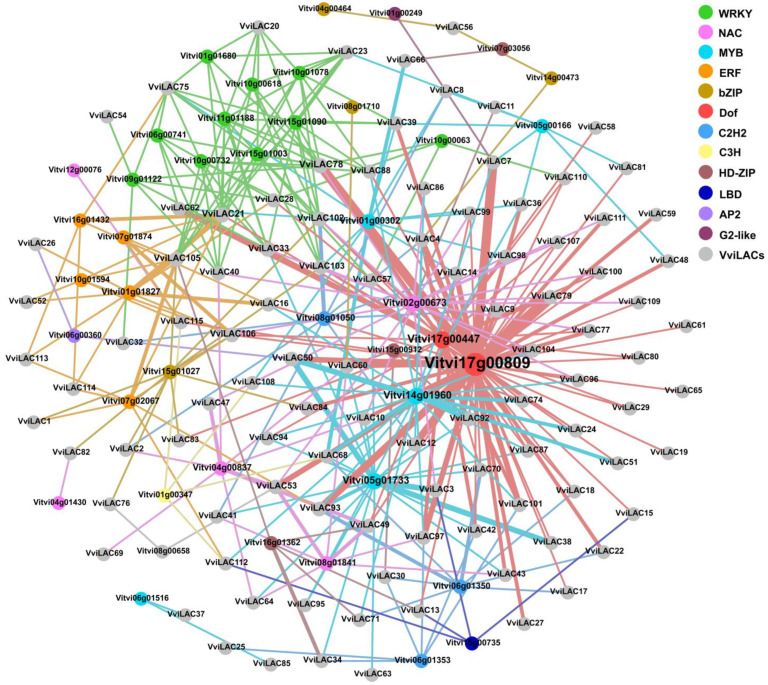
Transcription factors target the regulatory network of laccase genes. Different color circles represent different types of transcription factors, gray represents grape laccase family genes. The line coarseness is controlled by the predictive regulation threshold. The circle size is determined by the weight.

Prediction of transcription factor genes targeting members of laccase family in grape based on PlantTFDB database, search for threshold control to 10^−5^ to predict regulatory transcription factors in the VviLACs promoter region (2000 bp upstream Table S3). To further narrow the scope of possible regulation, we screened the differentially expressed transcription factors in the prediction results and drew a network of transcription factors regulating *VviLACs* (Figure 7). Twelve possible transcription factors were predicted to be involved in regulation. The middle position of network regulation and the thickness of the connection showed the criticality of *Vitvi17g00809*/Dof, *Vitvi17g00447*/Dof, *Vitvi14g01960*/MYB, and *Vitvi05g01733*/MYB TFs. So, these genes may be candidate-targeted TFs for VviLACs. We surmise that WRKY and ERF transcription factors will regulate *VviLAC105*, and MYB and Dof transcription factors may regulate *VviLAC51*.

## 3. Discussion

Laccase is a multicopper oxidase that plays an important role in lignin biosynthesis and is involved in plant development and various stress responses. Many plant laccase gene families have been systematically analyzed [28]. As an important fruit and wine raw material, the study of the grape laccase gene is of great significance. However, little information is available about the *VviLACs* gene, especially studies related to secondary metabolism. In this research, a total of 115 *VviLACs* were identified based on the grape genome, and the VviLAC gene family was comprehensively analyzed. The number of *VviLACs* in grapes was higher than in most other plants studied, including Arabidopsis [6], kiwi fruit [28], *Punica granatum* [29], cotton [7], *Eucalyptus grandis* [30], and others. Tandem and segmental duplication events are important aspects that decide the number of genes in a particular gene family. The expansion of the grape laccase gene family is mainly due to tandem duplication and an increase in gene duplication events. Our study shows that tandem duplication is the primary mechanism for the expansion of the grape laccase gene family. This process indicates that gene duplication events have facilitated the growth of the laccase gene family during evolution. Gene duplication not only increases the number of laccase genes but also may lead to the diversification of gene functions and enhance adaptability, enabling grapes to better cope with changing environmental conditions. The existence of multiple laccase genes allows grapes to perform various physiological processes, including lignin synthesis, metabolite accumulation, and antioxidant responses, to meet their adaptive needs in different environments. During growth, grapes face a variety of diseases, such as downy mildew and gray mold. The resistance conferred by laccase to these diseases may have been selected during species evolution, promoting the increase in the number of laccase genes. Additionally, the rich secondary metabolites found in grapes may also play a significant role in the expansion of the laccase gene family.

In grape, 107 *VviLACs* were unevenly distributed on 16 chromosomes, and 8 *VviLACs* were located on unassigned contigs, among which a large range of laccase gene replication appeared on chromosome 18, which may be a special phenomenon in the process of species evolution [31] (Figure 2). The studies showed some important genes that are crucial for yield [32], berry size [33], seedless [32], and disease resistance [34] are located on chromosome 18 [35]. Laccase genes are the regulators of lignin-related pathways, so they are important for fruit development and yield in grapes [36]. The berry shape and yield are highly regulated by lignin units. The deposition of lignin monomers is an important phenomenon that regulates disease resistance, berry weight, and other berry characteristics [37]. In the process of grape berry development, the expression trends of laccase genes showed significant differences. During fruit development, acidity, sugar accumulation, and pigment changes are regulated by endogenous hormones such as gibberellin and ethylene [38]. The upregulation or downregulation of related laccase genes is closely linked to changes in these hormone levels [15]. Additionally, transcription factors play an important role in regulating the expression of laccase genes. During fruit development, specific transcription factors can regulate the expression of laccases to help the plant adapt to environmental changes. It is worth noting that at different stages of fruit development, some laccase genes may show stage-specific expression patterns. A similar pattern in gene expression was reported in apples, where *LAC* genes were upregulated during later stages of apple fruit development [39]. The late stages of fruit development include the deposition of lignin monomers. A similar pattern was observed in ‘Summer black’ in our RT-qPCR test. Moreover, *LAC1, 2, 3, 90,* and *110* genes in *P. trichocarpa* showed higher expression during the lignin deposition stage [4].

All 115 *VviLACs* possess conserved copper-binding domains, but they exhibit diverse gene structures, indicating similar genetic origins but distinct biological functions. Furthermore, to examine the correlation between laccase and secondary metabolites, we analyzed the expression profile of *VviLACs* in berry tissues throughout the grape berry development phases. We discovered that 29 *VviLACs* were expressed in fruits with unique expression patterns. Correlation analysis with metabolome data and gene expression data showed that *VviLACs* were indeed correlated with metabolites. Interestingly, the expressions of *VviLAC51* and *VviLAC6* decreased with fruit development, while the expressions of *VviLAC4*, *12*, *16,* and *18* peaked at the fruit color transition stage and then gradually decreased, while the expression of *VviLAC105* increased with fruit development.

Based on the sequence comparison results, a phylogenetic tree containing 17 *AtLACs* and 115 *VviLACs* was constructed, and 7 taxa were identified through phylogenetic analysis (Figure 1). It was proved that *AtLAC4*, *AtLAC11*, *AtLAC2*, and *AtLAC17* are related to lignin biosynthesis, indicating that *VviLACs* in groups V, VI, and VII may participate in lignin biosynthesis. In Group IV, four *VviLACs* were clustered with Arabidopsis laccases *AtLAC7*, *AtLAC8*, and *AtLAC9*, which respond to environmental signals. It is worth noting that in group III, the amount of *VviLACs* aggregated with *AtLAC14* and *AtLAC15* is very large, and *AtLAC14* and *AtLAC15* participate in the polymerization of phenolic compounds. The genes in group III are the main reason for the expansion of *VviLACs* in grapes. Moreover, most of the genes were located on chromosome 18, suggesting a special mechanism of action for *VviLACs* in the polymerization of phenolic compounds. This may also be one of the key regulatory pathways for the accumulation of secondary metabolites in grape.

The overexpression of *LcLac* in the corpus callosum of litchi resulted in the overexpression of the laccase gene and the enhancement of laccase activity, while the content of polyphenols is decreased, thus accelerating the browning of the corpus callosum [40]. In contrast, in genetically modified plants of the *LcLac* gene knockdown with CRISPR/Cas9 technology, the polyphenol content showed the opposite trend, by delaying the browning [41]. This suggests the potential of laccase in the process of secondary metabolic accumulation. During grape berry development, *VviLACs* showed an association with secondary metabolites such as phenols, flavonoids, and alcohols. Especially *VviLAC51*, *53*, *60*, *62*, and *105* genes in group III. In addition, the transcription of the laccase gene is regulated by a variety of transcription factors, such as WRKY, MYB, NAC, ERF, etc. These transcription factors affect the transcription level of the laccase gene through specific binding to the promoter region of the laccase gene. At present, some transcription factors related to transcription regulation of laccase genes have been found [42]. For example, under light conditions, the photo-responsive factors *MdHY5*, *MdWRKY31,* and *MdLAC7* in apple showed ability to inhibit the transcription process and prevent peel Browning [43]. After light reduction (bagging treatment), the binding of *MdHY5* with *MdWRKY31* and *MdLAC7* was inhibited, and the transcriptional activities of *MdWRKY31* and *MdLAC7* were enhanced, and finally, the accumulation of phenolic substances and flavonoids was induced, which promoted the occurrence of peel Browning [43]. However, many potential regulatory factors, such as WRKY and MYB, have been found in the transcriptional regulatory network of *VviLACs*. To understand more regulatory mechanisms, more related molecular experiments are needed. These studies will help to elucidate the transcriptional regulatory network of laccase genes and provide a theoretical basis for the development of new regulatory strategies.

## 4. Materials and Methods

### 4.1. Grape Sampling and Developmental Stages

‘Summer black’ grape berries were collected from the grape research farm of National Grape Germplasm Repository at Zhengzhou Fruit Research Institute of the Chinese Academy of Agricultural Sciences. The sampling was performed in five stages according to the E-L system: EL-31 (pea-sized grape berries), EL-33 (hard and green grape berries), EL-34 (grape berries begin to soften), EL-35 (sugar start increasing), and EL-37 (berry not quite ripe) [27,44,45]. Five-year-old vines were randomly selected, and fruits with the same development status and size were collected at each time point. Samples were collected in triplicates. The berry samples at different stages were wrapped in aluminum foil, frozen in liquid nitrogen, and stored at −80 °C for later use.

### 4.2. Identification of VviLACs in the Grapevine Genome

To identify and annotate the laccase gene family in the grapevine genome, the grapevine laccase candidate genes were identified based on the HMM (Hidden Markov Model) profile of laccase (PF00394, PF07731, and PF07732) [46], with an e-value cutoff of 0.001 (https://urgi.versailles.inra.fr/Species/Vitis/Data-Sequences/Genome-sequences, accessed on 15 March 2024) [47] in grape genome. To avoid false laccase members because of incomplete Cu-oxidase domains, a BLASTP-algorithm-based search using 17 Arabidopsis laccase amino acid sequences as queries was conducted with an e-value ≤ 1 × 10^−5^. After eliminating redundancy, we obtained 119 potential laccase genes, which were then submitted to Pfam (https://www.ebi.ac.uk/interpro/, accessed on 12 January 2023) [48] and SMART (http://smart.embl-heidelberg.de/, accessed on 15 March 2024) [49] to identify the proteins containing the Cu-oxidase domain. By following these, a total of 115 *VviLACs* were identified and named based on their chromosomal positions. To further analyze these proteins, we utilized the Protparam online tools (https://web.expasy.org/protparam/, accessed on 15 March 2024) to predict their physicochemical properties. Additionally, the subcellular positions of the VviLACs proteins were predicted using Protcomp (http://www.softberry.com/, accessed on 10 January 2024).

### 4.3. Bioinformatics Analysis of Laccase Gene Family

CLUSTALX 2.0 software [50] was used to conduct a multiple-sequence alignment of 115 grapevine and 17 Arabidopsis laccase protein sequences. Additionally, any untrusted gaps at both ends of the sequences were manually eliminated using the same software. A phylogenetic tree was generated in MEGA 7.0 using the ML (maximum likelihood) method and bootstrapping with *n* = 1000 replicates, with default values [51]. The online EVOLVIEW (https://www.evolgenius.info/evolview/#login/, accessed on 22 March 2024) tool was used to carry out the tree visualization [52]. The online program Gene Structure Display Server 2.0 [53] was used to identify the genetic structure of the *VviLACs* (http://gsds.cbi.pku.edu.cn/, accessed on 22 March 2024). Using the MEME [54] online program (https://meme-suite.org/meme/tools/meme, accessed on 25 March 2024), the VviLACs protein sequences were analyzed to identify motifs. The parameters used for analysis were an optimal motif width ranging from 6 to 50 and a maximum number of ten motifs [55]. According to the annotated positions in grapevine genome data, TBtools (v1.087) software [56] was used for the mapping of *VviLACs* chromosomal positions and relative distances. The *VviLACs* gene duplication was confirmed based on two criteria: (1) the length of the shorter aligned sequence covered >75% of the longer sequence; and the similarity of the two aligned sequences was >75% [57]. (2) Two genes separated by five or fewer genes in a 100-kb chromosome fragment were considered tandemly duplicated genes [58]. MCScanX [59] program (sets the threshold to intercept 10^−5^) was used to detect the collinear region between *VviLACs* in *V. vinifera* L., *Oryza sativa* L., *A.s thaliana*, *Solanum lycopersicum*, *Gossypium raimondii*, *Populus trichocarpa*, and *Prunus persica* (the genome files of all species were downloaded from Ensembl: http://plants.ensembl.org/index.html, accessed on 3 April 2024). Regulatory cis-elements were analyzed in the promoter sequences of *VvLACs* within the 2000 bp region upstream of the start codon of each gene, using the Plant Cis-acting Regulatory Elements Database (http://bioinformatics.psb.ugent.be/webtools/plantcare/html/, accessed on 3 April 2024).

### 4.4. Correlation Analysis of VvLACs Expression and Secondary Metabolites

Based on previous studies of the transcriptome and metabolome during the growth and development of ‘Pinot Noir’ [27], the Pearson correlation between genes and metabolites was calculated using R-pack-psych. The image was drawn using the R package ggplot2.

### 4.5. RNA Extraction and Quantitative Real-Time PCR (qRT-PCR)

The qRT-PCR primers were designed using Primer Premier 5.0 software. The RNA from grapevine (*V. vinifera* cv. ‘Summer black’) berries was extracted with a Spectrum Plant Total RNA Kit (Sigma-Aldrich, Beijing, China), followed by reverse transcription of RNA into cDNA was carried out with the Prime Script RT Reagent Kit (Takara, Dalian, China). The qRT-PCR was performed in an IQ5 real-time PCR detection system (Bio-Rad Laboratories, Hercules, CA, USA) with SYBR Premium EX Taq II (Takara, Dalian, China). The total reaction volume was 25 μL. The relative expression level corresponding to the *β-TUB4* gene was calculated by using the 2^−ΔΔCt^ method [60]. Each reaction was prepared in triplicate and repeated three times. Primer sequence information is provided in Table S4.

### 4.6. Predictive Analysis of Candidate TFs Targeting Laccase Family Genes

Based on the GFF3 annotation file and the grape genome file [47], the promoter sequence of the grape laccase gene was extracted and submitted to PlantTFDB (http://plantregmap.gao-lab.org/regulation_prediction.php, accessed on 3 April 2024) for gene regulation prediction [61]. The sets of high-quality binding motifs of TFs and FIMO were used to scan TF binding sites in the promoters. The prediction information was obtained and imported into Gephi 0.9.2 software [62]. The “Fruchterman–Reingold” layout was used to visualize the data and calculate the average weight of the node to distinguish the criticality of the node.

### 4.7. Significance Analysis

SPSS version 21.0 was employed to analyze the statistically significant differences in the gene expression levels by ANOVA and Duncan’s multiple range test. All experiments were repeated three times as independent analyses.

## 5. Conclusions

The 115 *VviLAC* genes are mainly divided into 7 classes (type I–VII), distributed on 17 chromosomes. Of the 47 *VviLACs* on chromosome 18, 34 were involved in tandem repeat events, accounting for about 72.34%. Transcriptome data and qRT-PCR results showed that *VviLAC4, VviLAC12, VviLAC16, VviLAC18, VviLAC20, VviLAC53, VviLAC60,* and *VviLAC105* were highly expressed after fruit color transformation. *VviLAC105* was significantly positively correlated with key metabolites such as resveratrol, resveratrol dimer, paeoniflorin-3-glucoside, etc. The grapevine has a higher number of *VviLAC* genes, signifying their role in metabolite synthesis. Grapevine is known for metabolites that are beneficial to human health or the defense system of the plant itself. The transcriptional regulatory network prediction showed that twelve transcription factors targeted *VviLACs*, among these WRKY and ERF were potential transcriptional regulators of *VviLAC105*. Dof and MYB are potential transcriptional regulators of *VviLAC51*. The study of these genes will provide important information in the synthesis of secondary metabolites, which is important for any commercial use of grapevine. These compounds are a high-valued product in the wine industry.

## Data Availability

The grapevine berry RNA-Seq data accession number is GSE98923. The reference for the microarray data is GSE113225. The SubSeries (GSE113223and GSE113224). The source research is cited in the manuscript. The data can be downloaded from the NCBI official website using the SubSeries number.

## Supplementary Materials

The following supporting information can be downloaded at: https://www.mdpi.com/article/10.3390/ijms251910574/s1.

## References

[1] Bao W., O’Malley D.M., Whetten R., Sederoff R.R. (1993). A laccase associated with lignification in loblolly pine xylem. Science.

[2] Zhao Q., Nakashima J., Chen F., Yin Y., Fu C., Yun J., Shao H., Wang X., Wang Z.-Y., Dixon R.A. (2013). Laccase is necessary and nonredundant with peroxidase for lignin polymerization during vascular development in *Arabidopsis*. Plant Cell.

[3] Iqbal M.J., Ahsan R., Afzal A., Jamai A., Meksem K., El-Shemy H.A., Lightfoot D.A. (2009). Multigeneic QTL: The laccase encoded within the soybean Rfs2/rhg1 locus inferred to underlie part of the dual resistance to cyst nematode and sudden death syndrome. Curr. Issues Mol. Biol..

[4] Ranocha P., Chabannes M., Chamayou S., Danoun S., Jauneau A., Boudet A.-M., Goffner D. (2002). Laccase down-regulation causes alterations in phenolic metabolism and cell wall structure in poplar. Plant Physiol..

[5] McCaig B.C., Meagher R.B., Dean J.F. (2005). Gene structure and molecular analysis of the laccase-like multicopper oxidase (LMCO) gene family in *Arabidopsis thaliana*. Planta.

[6] Turlapati P.V., Kim K.-W., Davin L.B., Lewis N.G. (2011). The laccase multigene family in *Arabidopsis thaliana*: Towards addressing the mystery of their gene function (s). Planta.

[7] Balasubramanian V.K., Rai K.M., Thu S.W., Hii M.M., Mendu V. (2016). Genome-wide identification of multifunctional laccase gene family in cotton (*Gossypium* spp.); expression and biochemical analysis during fiber development. Sci. Rep..

[8] Liu Q., Luo L., Wang X., Shen Z., Zheng L. (2017). Comprehensive analysis of rice laccase gene (OsLAC) family and ectopic expression of OsLAC10 enhances tolerance to copper stress in *Arabidopsis*. Int. J. Mol. Sci..

[9] Xu X., Zhou Y., Wang B., Ding L., Wang Y., Luo L., Zhang Y., Kong W. (2019). Genome-wide identification and characterization of laccase gene family in *Citrus sinensis*. Gene.

[10] Simões M.S., Carvalho G.G., Ferreira S.S., Hernandes-Lopes J., de Setta N., Cesarino I. (2020). Genome-wide characterization of the laccase gene family in Setaria viridis reveals members potentially involved in lignification. Planta.

[11] Ping X., Wang T., Lin N., Di F., Li Y., Jian H., Wang H., Lu K., Li J., Xu X. (2019). Genome-wide identification of the LAC gene family and its expression analysis under stress in *Brassica napus*. Molecules.

[12] Cheng X., Li G., Ma C., Abdullah M., Zhang J., Zhao H., Jin Q., Cai Y., Lin Y. (2019). Comprehensive genome-wide analysis of the pear (*Pyrus bretschneideri*) laccase gene (PbLAC) family and functional identification of PbLAC1 involved in lignin biosynthesis. PLoS ONE.

[13] Berthet S., Demont-Caulet N., Pollet B., Bidzinski P., Cézard L., Le Bris P., Borrega N., Hervé J., Blondet E., Balzergue S. (2011). Disruption of LACCASE4 and 17 results in tissue-specific alterations to lignification of *Arabidopsis thaliana* stems. Plant Cell.

[14] Jeon J.R., Baldrian P., Murugesan K., Chang Y.S. (2012). Laccase-catalysed oxidations of naturally occurring phenols: From in vivo biosynthetic pathways to green synthetic applications. Microb. Biotechnol..

[15] Hu Q., Min L., Yang X., Jin S., Zhang L., Li Y., Ma Y., Qi X., Li D., Liu H. (2018). Laccase GhLac1 modulates broad-spectrum biotic stress tolerance via manipulating phenylpropanoid pathway and jasmonic acid synthesis. Plant Physiol..

[16] Pourcel L., Routaboul J.-M., Kerhoas L., Caboche M., Lepiniec L., Debeaujon I. (2005). TRANSPARENT TESTA10 encodes a laccase-like enzyme involved in oxidative polymerization of flavonoids in *Arabidopsis* seed coat. Plant Cell.

[17] Liang M., Davis E., Gardner D., Cai X., Wu Y. (2006). Involvement of AtLAC15 in lignin synthesis in seeds and in root elongation of *Arabidopsis*. Planta.

[18] Fang F., Zhang X.-L., Luo H.-H., Zhou J.-J., Gong Y.-H., Li W.-J., Shi Z.-W., He Q., Wu Q., Li L. (2015). An intracellular laccase is responsible for epicatechin-mediated anthocyanin degradation in litchi fruit pericarp. Plant Physiol..

[19] Yadav V., Wang Z., Wei C., Amo A., Ahmed B., Yang X., Zhang X. (2020). Phenylpropanoid Pathway Engineering: An Emerging Approach towards Plant Defense. Pathogens.

[20] Bagal U.R., Leebens-Mack J.H., Lorenz W.W., Dean J.F.D. (2012). The phenylalanine ammonia lyase (PAL) gene family shows a gymnosperm-specific lineage. BMC Genom..

[21] Wang W., Wang C., Xie Z., Jia H., Tang W., Cui M., Fang J. (2018). Function analysis of VvmiR397a and its target genes VvLACs in grape berry development. Acta Hortic. Sin..

[22] Huang Y., Liang D., Xia H., Lin L.-J., Wang J., Lv X.-L. (2020). Lignin and Quercetin Synthesis Underlies Berry Russeting in ‘Sunshine Muscat’ Grape. Biomolecules.

[23] Aruni U.V. (2017). Role of Laccase as a Virulence Factor in the Infection of Grapes by *Botrytis cinerea*. Doctoral Thesis.

[24] Wang G.-D., Li Q.-J., Luo B., Chen X.-Y. (2004). Ex planta phytoremediation of trichlorophenol and phenolic allelochemicals via an engineered secretory laccase. Nat. Biotechnol..

[25] Cho H.Y., Lee C., Hwang S.-G., Park Y.C., Lim H.L., Jang C.S. (2014). Overexpression of the OsChI1 gene, encoding a putative laccase precursor, increases tolerance to drought and salinity stress in transgenic *Arabidopsis*. Gene.

[26] Ma J., Xu Z.-S., Wang F., Xiong A.-S. (2015). Isolation, purification and characterization of two laccases from carrot (*Daucus carota* L.) and their response to abiotic and metal ions stresses. Protein J..

[27] Fasoli M., Richter C.L., Zenoni S., Bertini E., Vitulo N., Dal Santo S., Dokoozlian N., Pezzotti M., Tornielli G.B. (2018). Timing and Order of the Molecular Events Marking the Onset of Berry Ripening in Grapevine. Plant Physiol..

[28] Zhang Z., Long Y., Yin X., Wang W., Li W., Jiang L., Chen X., Wang B., Ma J. (2023). Genome-wide identification and expression patterns of the laccase gene family in response to kiwifruit bacterial canker infection. BMC Plant Biol..

[29] Shi J., Yao J., Tong R., Wang S., Li M., Song C., Wan R., Jiao J., Zheng X. (2023). Genome-wide identification of laccase gene family from Punica granatum and functional analysis towards potential involvement in lignin biosynthesis. Horticulturae.

[30] Arcuri M.L., Fialho L.C., Vasconcellos Nunes-Laitz A., Fuchs-Ferraz M.C.P., Wolf I.R., Valente G.T., Marino C.L., Maia I.G. (2020). Genome-wide identification of multifunctional laccase gene family in Eucalyptus grandis: Potential targets for lignin engineering and stress tolerance. Trees.

[31] Qiao X., Li Q., Yin H., Qi K., Li L., Wang R., Zhang S., Paterson A.H. (2019). Gene duplication and evolution in recurring polyploidization–diploidization cycles in plants. Genome Biol..

[32] Cabezas J.A., Cervera M.T., Ruiz-García L., Carreño J., Martínez-Zapater J.M. (2006). A genetic analysis of seed and berry weight in grapevine. Genome.

[33] Doligez A., Bouquet A., Danglot Y., Lahogue F., Riaz S., Meredith C., Edwards K., This P. (2002). Genetic mapping of grapevine (*Vitis vinifera* L.) applied to the detection of QTLs for seedlessness and berry weight. Theor. Appl. Genet..

[34] Bellin D., Peressotti E., Merdinoglu D., Wiedemann-Merdinoglu S., Adam-Blondon A.-F., Cipriani G., Morgante M., Testolin R., Di Gaspero G. (2009). Resistance to Plasmopara viticola in grapevine ‘Bianca’is controlled by a major dominant gene causing localised necrosis at the infection site. Theor. Appl. Genet..

[35] Töpfer R., Hausmann L., Harst M., Maul E., Zyprian E., Eibach R. (2011). New horizons for grapevine breeding. Fruit Veg. Cereal Sci. Biotechnol..

[36] Fernandes J.C., Goulao L.F., Amâncio S. (2016). Regulation of cell wall remodeling in grapevine (*Vitis vinifera* L.) callus under individual mineral stress deficiency. J. Plant Physiol..

[37] Malacarne G., Lagreze J., Rojas San Martin B., Malnoy M., Moretto M., Moser C., Dalla Costa L. (2024). Insights into the cell-wall dynamics in grapevine berries during ripening and in response to biotic and abiotic stresses. Plant Mol. Biol..

[38] Schwab W., Raab T. (2004). Developmental changes during strawberry fruit ripening and physico-chemical changes during postharvest storage. Production Practices and Quality Assessment of Food Crops: Quality Handling and Evaluation.

[39] Yihui G., Song J., Du L., Vinqvist M., Palmer L.C., Fillmore S., Pang X., Zhang Z. (2018). Characterization of laccase from apple fruit during postharvest storage and its response to diphenylamine and 1-methylcyclopropene treatments. Food Chem..

[40] Liu B., Zhong R., Wei J., Zhang J., Luo H., Guan H., Fang F., Pang X., Zhang Z. (2024). Genome-wide identification and analysis of the laccase gene family in *Litchi chinensis* Sonn. provides new insights into pericarp browning. Postharvest Biol. Technol..

[41] Zhou Z., Li Q., Xiao L., Wang Y., Feng J., Bu Q., Xiao Y., Hao K., Guo M., Chen W. (2021). Multiplexed CRISPR/Cas9-Mediated Knockout of Laccase Genes in Salvia miltiorrhiza Revealed Their Roles in Growth, Development, and Metabolism. Front. Plant Sci..

[42] Zhang L.B., Yang W.W.J., Qiu T.T. (2023). Genome-wide study of *Cerrena unicolor* 87613 laccase gene family and their mode prediction in association with substrate oxidation. BMC Genomics.

[43] Wang H., Zhang S., Fu Q., Wang Z., Liu X., Sun L., Zhao Z. (2023). Transcriptomic and metabolomic analysis reveals a protein module involved in preharvest apple peel browning. Plant Physiol..

[44] Wang H., Liu C., Sun L., Yang S., Fan X., Zhang Y., Guo D., Jiang J. (2023). RNA-sequencing analysis of candidate genes involved in berry development in ‘Summer Black’ grapes and its early bud mutants varieties. Sci. Hortic..

[45] Dry P., Coombe B. (2004). Revised Version of “Grapevine Growth Stages—The Modified el System” Viticulture 1.

[46] Finn R.D., Clements J., Eddy S.R. (2011). HMMER web server: Interactive sequence similarity searching. Nucleic Acids Res..

[47] Canaguier A., Grimplet J., Di Gaspero G., Scalabrin S., Duchêne E., Choisne N., Mohellibi N., Guichard C., Rombauts S., Le Clainche I. (2017). A new version of the grapevine reference genome assembly (12X. v2) and of its annotation (VCost. v3). Genom. Data.

[48] El-Gebali S., Mistry J., Bateman A., Eddy S.R., Luciani A., Potter S.C., Qureshi M., Richardson L.J., Salazar G.A., Smart A. (2019). The Pfam protein families database in 2019. Nucleic Acids Res..

[49] Letunic I., Doerks T., Bork P. (2012). SMART 7: Recent updates to the protein domain annotation resource. Nucleic Acids Res..

[50] Larkin M.A., Blackshields G., Brown N.P., Chenna R., McGettigan P.A., McWilliam H., Valentin F., Wallace I.M., Wilm A., Lopez R. (2007). Clustal W and Clustal X version 2.0. Bioinformatics.

[51] Kumar S., Stecher G., Tamura K. (2016). MEGA7: Molecular evolutionary genetics analysis version 7.0 for bigger datasets. Mol. Biol. Evol..

[52] Zhang H., Gao S., Lercher M.J., Hu S., Chen W.-H. (2012). EvolView, an online tool for visualizing, annotating and managing phylogenetic trees. Nucleic Acids Res..

[53] Hu B., Jin J., Guo A.-Y., Zhang H., Luo J., Gao G. (2015). GSDS 2.0: An upgraded gene feature visualization server. Bioinformatics.

[54] Bailey T.L., Johnson J., Grant C.E., Noble W.S. (2015). The MEME suite. Nucleic Acids Res..

[55] Ji X.R., Yu Y.H., Ni P.Y., Zhang G.H., Guo D.L. (2019). Genome-wide identification of small heat-shock protein (HSP20) gene family in grape and expression profile during berry development. BMC Plant Biol..

[56] Chen C., Chen H., Zhang Y., Thomas H., Frank M., He Y., Xia R. (2020). TBtools—An Integrative Toolkit Developed for Interactive Analyses of Big Biological Data. Mol. Plant.

[57] Ni P., Ji X., Guo D. (2020). Genome-wide identification, characterization, and expression analysis of GDSL-type esterases/lipases gene family in relation to grape berry ripening. Sci. Hortic..

[58] Zhao P., Wang D., Wang R., Kong N., Zhang C., Yang C., Wu W., Ma H., Chen Q. (2018). Genome-wide analysis of the potato Hsp20 gene family: Identification, genomic organization and expression profiles in response to heat stress. BMC Genom..

[59] Wang Y., Tang H., DeBarry J.D., Tan X., Li J., Wang X., Lee T.-H., Jin H., Marler B., Guo H. (2012). MCScanX: A toolkit for detection and evolutionary analysis of gene synteny and collinearity. Nucleic Acids Res..

[60] Livak K.J., Schmittgen T. (2001). Analysis of relative gene expression data using real-time quantitative PCR and the 2(-Delta Delta C(T)) Method. Methods.

[61] Jin J., Tian F., Yang D.-C., Meng Y.-Q., Kong L., Luo J., Gao G. (2016). PlantTFDB 4.0: Toward a central hub for transcription factors and regulatory interactions in plants. Nucleic Acids Res..

[62] Bastian M., Heymann S., Jacomy M. Gephi: An open source software for exploring and manipulating networks. Proceedings of the International AAAI Conference on Web and Social Media.

