# Supplementary figures and images for: Identification of Grape Laccase Genes and Their Potential Role in Secondary Metabolite Synthesis
Source: Int J Mol Sci. 2024 Sep 30;25(19):10574. doi: 10.3390/ijms251910574 (PMC11476532; doi:10.3390/ijms251910574)

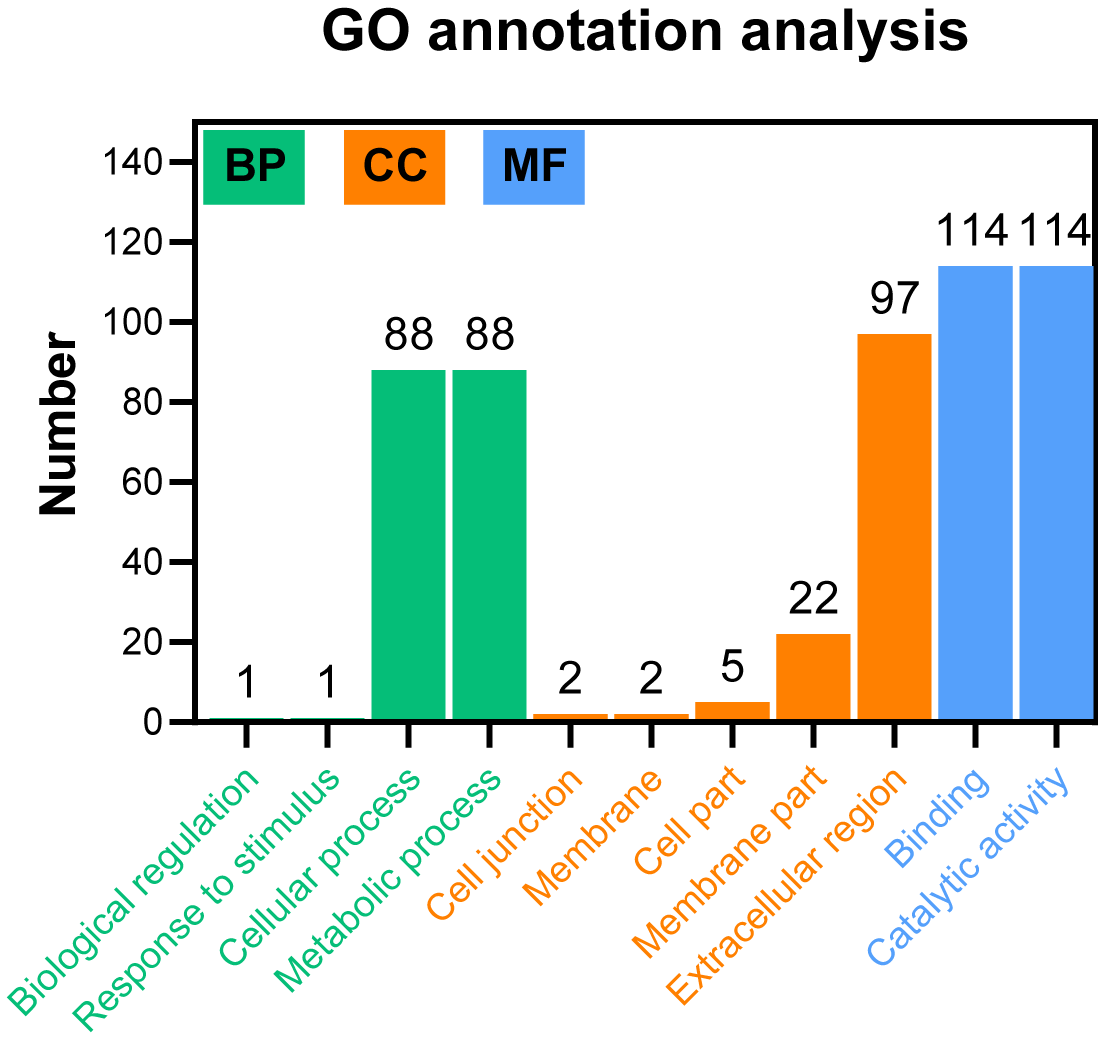

Supplement: Supplementary file 1 [file ijms-25-10574-s001.zip › Figure S1.tif]
